# A Real-Time Computer-Aided Diagnosis System for Coronary Heart Disease Prediction Using Clinical Information

**DOI:** 10.31083/RCM26204

**Published:** 2025-03-17

**Authors:** Huiqian Tao, Chengfeng Wang, Hongxia Qi, Hui Li, Yane Li, Ruifei Xie, Yuzhu Dai, Qingyang Sun, Yingqiang Zhang, Xinyi Yu, Tingting Shen

**Affiliations:** ^1^Department of Clinical Research, The 903rd Hospital of The People’s Liberation Army, 310013 Hangzhou, Zhejiang, China; ^2^College of Mathematics and Computer Science, Zhejiang A&F University, 311300 Hangzhou, Zhejiang, China; ^3^Department of Echocardiography, Fuwai Hospital, National Center for Cardiovascular Diseases, Chinese Academy of Medical Sciences and Peking Union Medical College, 100037 Beijing, China; ^4^Information Department, Hangzhou Cancer Hospital, 310000 Hangzhou, Zhejiang, China

**Keywords:** coronary heart disease, prediction model, real-time, machine learning, singular value decomposition

## Abstract

**Background:**

It is important to establish a coronary heart disease (CHD) prediction model with high efficiency and precision for early diagnosis of CHD using clinical information. While existing deep learning-based CHD prediction models possess the limitations of large datasets and long training time, existing machine learning-based CHD prediction models have the limitations of low accuracy and robustness, which are unsuitable for clinical application. This study aimed to design a fast and high-precision intelligent model using clinical information to predict CHD.

**Methods:**

Five public datasets, including 303, 293, 303, 200, and 123 patients with 55, 14, 14, 14, and 14 attributes, respectively, were used for model training and testing. After data preprocessing, the singular value decomposition method was utilized to extract features to build the CHD prediction model. Then, the CHD prediction model was established using the 5-fold cross-validation method with a multilayer perceptron approach.

**Results:**

Results show that the established model performs better on the total dataset than the other models we built in this study. This machine learning-based CHD prediction model achieved an improved area under the curve (AUC*)* of 99.10%, with 96.63% accuracy, 96.50% precision, 97.4% recall, and 97.0% *F*_1_-score on the total dataset.

**Conclusions:**

This high precision and efficiency achieved by the proposed model on different datasets would be significant for the prediction of CHD for medical and clinical diagnosis purposes.

## 1. Introduction

Coronary heart disease (CHD) is one of the leading causes of illness and death worldwide. According to the American Heart Association, the prevalence of cardiovascular diseases, including coronary heart disease, heart failure, stroke, and hypertension, among adults whose age is higher than 20 years in the United States is as high as 49.2%, affecting approximately 126.9 million individuals in 2018 [1]. In 2019, CHD was the leading cause of cardiovascular disease (CVD)-related deaths in the United States, accounting for 41.3% [1]. The number of CHD cases will be increased by nearly 100 percent in 2030, predicted by the American Heart Association [2]. The high incidence of CHD places a huge burden on society and healthcare resources [3]. Early diagnosis and treatment are essential to reduce cardiac arrest and mortality in patients with CHD [3]. The techniques currently used to predict and diagnose heart disease are largely based on analyses of the patient’s medical history, symptoms, and physical examination reports. However, the predictive accuracy is only about 67% [4]. It is important to develop a more precise and efficient approach to predict CHD promptly at an early stage [5]. Over the past few decades, many researchers have focused on developing different predictive models and methods with biomarkers, genetic information, medical imaging, and clinical features to identify the risk of CHD. The details are as follows.

Biomarkers-based CHD prediction method. A CHD prediction model established using the identified CHD-related biomarkers is a common CHD assessment approach that can help monitor and prevent CHD risk. For example, Costa *et al*. [6] performed a case–control study to predict the prevalence of CHD by circulating non-coding small RNA sRNY1-5p, which indicated that the serum s-RNY1-5p was an independent predictor for CHD events in the general male population [6]. Qi *et al*. [7] proposed a CHD assessment method by detecting plasma inflammatory cytokines, which showed that tumor necrosis factor (TNF) and heterogenous nuclear ribonucleoprotein L (hnRNPL) related immunoregulatory long non-coding RNA (*THRIL*) was increased in CHD patients and can be used to predict CHD risk. Ong *et al*. [8] measured 184 CHD-related biomarkers at the plasma level with the proximity extension method, which showed that collagen type I alpha 1 (*COL1A1*), bone morphogenetic protein 6 (*BMP-6*), and interleukin-6 receptor subunit alpha (*IL-6Rα*) were biomarkers in the development of CHD. Biomarkers-based methods can predict CHD, but selecting and collecting CHD-related biomarkers is difficult. In addition, the interpretability and generalizability of the biomarker-based CHD prediction models remain challenging.

The genetic information-based CHD prediction method is important in heart disease prediction. Researchers have analyzed genetic markers such as genetic variants, polymorphisms, and genomics associations to find CHD-related genetic factors. For example, Yun *et al*. [9] established a genetic risk score (GRS) weighted by 55 single nucleotide polymorphisms, showing that GRS can improve the accuracy of CHD prediction in the male population. Nasr *et al*. [10] combined genetic and non-genetic factors to establish a CHD prediction model. Dogan *et al*. [11] developed an integrated genetic epigenetic biomarker model to predict CHD risk within the next three years. Zhang *et al*. [12] proposed a deep learning-based CHD prediction model with gene expression data, inter-gene interaction information, and reported susceptibility loci. Bauer *et al*. [13] compared and analyzed the effectiveness of various genetic risk-based CHD prediction models. However, obtaining and analyzing genetic information requires large-scale genetic data with sophisticated statistical methods and consideration of the influence of non-genetic factors such as environment and lifestyle.

The medical imaging-based CHD prediction method is an important predictive tool. Images, including electrocardiograms, echocardiograms, and magnetic resonance imaging (MRI) images, can describe detailed information about the structure and function of the heart and can provide strong support for the prediction of heart disease. For example, Haji *et al*. [14] performed a randomized controlled trial by analyzing echocardiograms of patients, which demonstrated that global longitudinal strain was independently associated with the risk of CHD. Dutta *et al*. [15] developed a convolutional neural network architecture to predict the development of CHD, which achieved an accuracy value of 77% on the National Health and Nutrition Examination Survey dataset. Denzinger *et al*. [16] evaluated the performance of a deep learning-based CHD assessment model established using computed tomography (CT) images. However, the medical imaging-based CHD prediction method is limited in expertise and highly complex algorithms, large data volumes, and noise interference.

The clinical characteristics-based CHD prediction method has recently been established using clinical characteristics, including patients’ personal and medical information, and has attracted increased attention. Recent advances in machine learning techniques provide research opportunities in the early prediction of cardiovascular disease to improve patient survival [17, 18]. For example, Fan *et al*. [19] implemented a quantitative model to identify the ones with high risks of CHD complications for type 2 diabetes mellitus patients by combining random forest and information entropy, which has an area under the curve (AUC) value of 0.77 on the training dataset and 0.80 on the test dataset [19]. Ayon *et al*. [20] compared several computational intelligence techniques, including logistic regression (LR), support vector machine (SVM), deep neural network (DNN), decision tree (DT), naïve Bayes (NB), random forest (RF), and k-nearest neighbors (kNN), in predicting coronary artery heart disease using the TATLOG and Cleveland Heart Disease datasets. Here, deep neural networks obtained the highest performance, with an accuracy value of 98.15%, a sensitivity value of 98.67%, and a precision value of 98.01%. Amarbayasgalan *et al*. [21] presented a deep neural network-based CHD risk prediction model using well-ordered datasets, which showed that the proposed method outperforms traditional machine learning algorithms with an accuracy value of 89.2% , a specificity value of 84.0% , and an AUC value of 88.2% . Mosley *et al*. [22] proposed an effective convolutional neural network for CHD prediction, which achieved an accuracy of 77% on cases with CHD and 81.8% on cases without CHD. Ananey-Obiri and Sarku [23] developed three models using LR, DT, and Gaussian plain Bayes (GNB), respectively, to predict heart disease using the Cleveland dataset, which showed that both LR and GNB achieved an accuracy of 82.75% with an AUC of 87.0%. Napa *et al*. [24] trained and compared five machine learning classifiers, i.e., LR, SVM, DT, RF, and kNN, on the University of California Irvine (UCI) dataset with 303 cases and 10 attributes to predict cardiovascular diseases. Results showed that the RF classifier achieved the highest performance with an accuracy of 85.71% and an AUC of 86.75% [24]. Perumal and Kaladevi [25] developed heart disease prediction models on the Cleveland dataset, which showed that LR and SVM-based models achieved accuracy values of 87% and 85%, respectively. In contrast, the kNN-based model reached an accuracy of 69%. Mohan *et al*. [26] developed a hybrid random forest with a linear model (HRFLM) to predict heart disease using the Cleveland dataset, which achieved an accuracy of 88.7%. Pavithra and Jayalakshmi [27] proposed a new hybrid feature selection technique combining random forest, AdaBoost, and linear correlation (HRFLC) to predict heart disease. Results show a 2% improvement in the accuracy of the hybrid model compared to the single model [27]. Trigka and Dritsas [28] used synthetic minority over-sampling technique (SMOTE) and a stacked ensemble learning model to predict long-term computer-aided diagnosis (CAD) with clinical information, which achieved a recall of 87.6% and an AUC value of 96.1%. Enad and Mohammed [29] proposed a cloud computing-based framework for heart disease classification using the quantum SVM method, which showed an accuracy of 85%, a precision of 79%, and a recall of 90%. Sarra *et al*. [30] proposed two DL-based frameworks, GAN-1D-CNN (generative adversarial network-a one-dimensional convolutional neural network) and GAN-Bi-LSTM (generative adversarial network-bidirectional long short-term memory), to predict heart disease, which achieved an accuracy of 99.1% and 99.3%, respectively. Sarra *et al*. [31] also proposed a heart disease prediction model using a support vector machine combined with χ^2^ statistical optimum feature selection technique, which achieved an accuracy of 89.7%. Despite the achievements of the above methods in heart disease prediction, some challenges and limitations remain [32, 33]. On the one hand, machine learning-based CHD prediction models need to process the high-dimensional data and select features, which remain challenging and affect the performance and generalization ability of the model. On the other hand, the dataset is small and unsuitable for deep learning-based CHD detection models, which need big data to train many parameters. Thus, it is important to research and advance AI-based approaches further to diagnose and manage heart diseases [34].

**Fig. 1.**
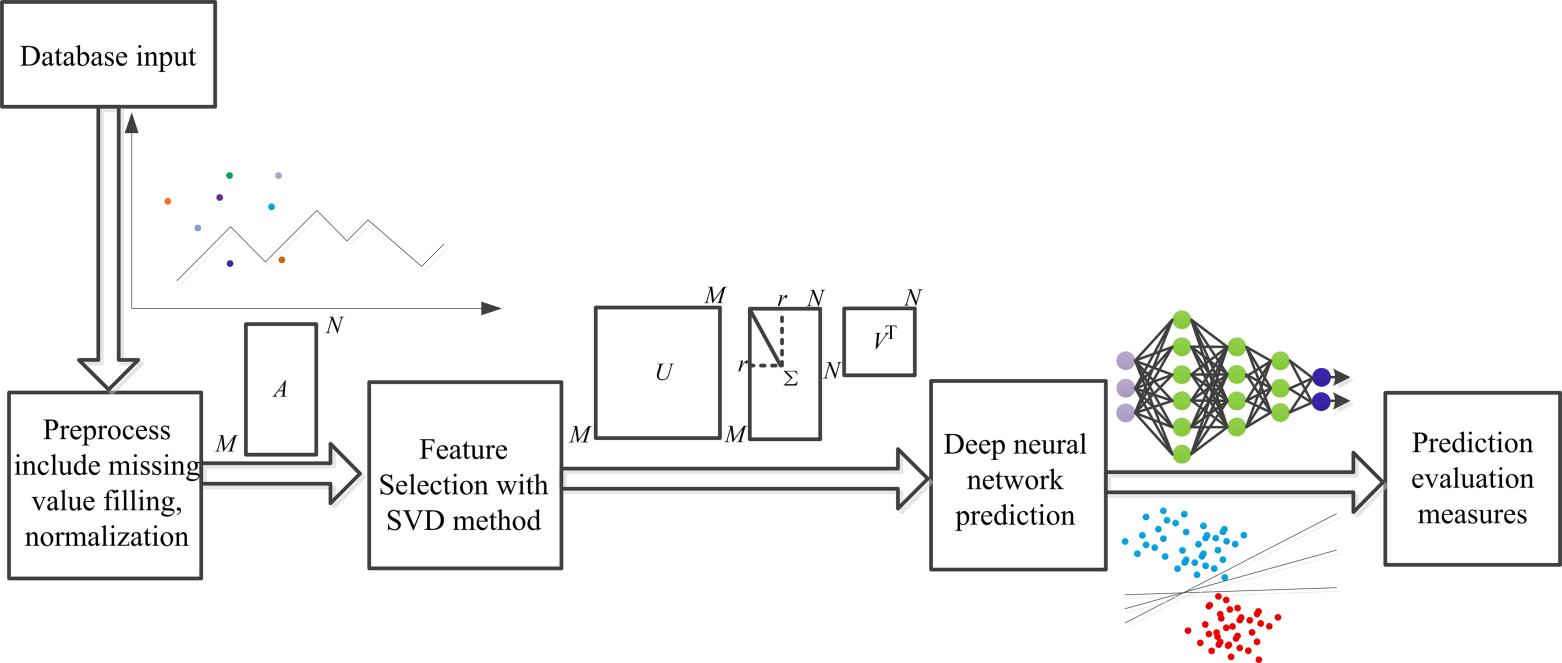
**General framework outline**. SVD, singular value decomposition.

These studies showed the effectiveness of the machine learning method using clinical information to predict CHD. However, the accuracy and robustness must be improved to satisfy clinical application requirements. The singular value decomposition (SVD) method is suitable for numerical data since it can reduce the dimensionality of the data and remove noise to improve the performance of the algorithm. In addition, the multilayer perceptron (MLP) method has strong nonlinear modeling ability, enabling it to learn more complex feature representations suitable for classification and regression tasks. Therefore, in this study, we propose a high-accuracy and robust CHD prediction model tested on five public datasets and one mixed dataset, as shown in Fig. 1.

Specifically, the SVD method was used for feature extraction after data preprocessing. Then, a CHD prediction model was established with a 5-fold cross-validation method using a MLP classifier. In addition, the other four CHD prediction models were also established with random tree, random forest, logistic, and MLP only. Finally, performance indices, including accuracy, precision, recall, *F*_1_-score, and AUC, were computed and compared for each model established in this study on five public and one mixed dataset, respectively. Results show that each performance index of this proposed model achieved the best compared to other models on each dataset tested in this study.

The primary contributions of this study are as follows:

- We developed a real-time applicable and high-performance CHD detection CAD model using clinical information. The developed CHD detection model represents a highly effective work that applies clinical information with a machine learning-based method, making it faster and perform better than other CHD detection models on multiple datasets.
- The performance of the developed CHD detection model was analyzed and compared by experimenting on five independent datasets with (i) different classifiers using features selected with SVD and (ii) different features including all attributes, principal component analysis (PCA)-selected features, and SVD-selected features with the MLP classifier. The SVD and MLP-supported CHD detection model was compared against other recent works using the same dataset. The proposed model achieved the highest AUC of 98.2%, along with an accuracy of 94.8% with a time of 0.24 seconds on the Z-Alizadeh Sani dataset, while an AUC of 99.1% and an accuracy of 96.63% were achieved for a hybrid dataset consisting of four independent datasets.
- The proposed model allows different sizes of feature vectors, which overcomes the challenge of irregularity in the data shape of clinical information, is applicable for massive data, and improves the model’s performance.
- The features responsible for diagnosis prediction can be accessed, visualized, and analyzed in the proposed intelligent system. In addition, these features will aid medical professionals in performing diagnoses quickly.

This paper is organized as follows: The first section is “Introduction”, which summarizes the background and significance of building a high-performance CHD detection model, the outline of the CHD detection method, recent advancements and limitations of CHD detection model, the method proposed in this paper, the main contributions of this paper, and the structure of this paper. The second section is “Materials and Methods”, which introduces the dataset information used in this paper, the SVD and MLP methods, and the model training and evaluation methods. The third section is “Results and Analysis”, which includes the performance and comparative analysis of the model constructed by our proposed method and another method on a single dataset and mixed datasets, and the performance and comparative analysis of models built with or without the SVD method on each dataset. The fourth section, “Discussion”, discusses the content of this paper in detail and summarizes the limitations of the method and possible future research methods. The fifth section, “Conclusions”, outlines the content of this article.

## 2. Materials and Methods

### 2.1 Dataset

Five public datasets, including the Z-Alizadeh Sani dataset [35], the Hungary dataset [36], the Cleveland dataset [36], the Long Beach–Virginia dataset [36], and Switzerland datasets [36], which contain 303, 293, 303, 200, and 123 patients, respectively, with 54, 14, 14, 14 and 14 attributes, respectively, were collected and used in this study. For the Z-Alizadeh Sani dataset, 54 clinical features were divided into four groups: demographics, symptoms and examination, electrocardiogram (ECG), laboratory, and echo features, as shown in Table 1.

**Table 1.** **Features of Z-Alizadeh Sani dataset**.

	Attributes	Type	Value
1	Age	Integer	(30–86)
2	Weight	Integer	(48–120)
3	Length	Integer	(140–188)
4	Sex	Integer	Male = 1; female = 0
5	BMI (body mass index) (kg/m^2^)	Fioat	(18.11–40.90)
6	DM (diabetes mellitus)	Integer	True = 1; false = 0
7	HTN (hypertension)	Integer	True = 1; false = 0
8	Current smoker	Integer	True = 1; false = 0
9	Ex-smoker	Integer	True = 1; false = 0
10	FH (family history)	Integer	True = 1; false = 0
11	Obesity	Integer	Yes = 1; no = 0
12	CRF (chronic renal failure)	Integer	Yes = 1; no = 0
13	CVA (cerebrovascular accident)	Integer	Yes = 1; no = 0
14	Airway disease	Integer	Yes = 1; no = 0
15	Thyroid disease	Integer	Yes = 1; no = 0
16	CHF (congestive heart failure)	Integer	Yes = 1; no = 0
17	DLP (dyslipidemia)	Integer	Yes = 1; no = 0
18	BP (blood pressure) (mmHg)	Integer	(90–190)
19	PR (pulse rate) (ppm)	Integer	(50–110)
20	Edema	Integer	True = 1; false = 0
21	Weak peripheral pulse	Integer	Yes = 1; no = 0
22	Lung rales	Integer	Yes = 1; no = 0
23	Systolic murmur	Integer	Yes = 1; no = 0
24	Diastolic murmur	Integer	Yes = 1; no = 0
25	Typical chest pain	Integer	True = 1; false = 0
26	Dyspnea	Integer	Yes = 1; no = 0
27	Function class	Integer	(0–3)
28	Atypical	Integer	Yes = 1; no = 0
29	Nonanginal	Integer	Yes = 1; no = 0
30	Exertional CP (exertional chest pain)	Integer	Yes = 1; no = 0
31	Low Th Ang (low threshold angina)	Integer	Yes = 1; no = 0
32	Q wave	Integer	True = 1; false = 0
33	St. elevation	Integer	True = 1; false = 0
34	St. depression	Integer	True = 1; false = 0
35	T wave inversion	Integer	True = 1; false = 0
36	LVH (left ventricular hypertrophy)	Integer	Yes = 1; no = 0
37	Poor R progression	Integer	Yes = 1; no = 0
38	BBB (blood–brain barrier)	Integer	LBBB = 1; RBBB = 1; no = 0
39	FBS (fasting blood sugar) (mg/dL)	Integer	(62, 400)
40	Cr (creatine) (mg/dL)	Float	(0.5, 2.2)
41	TG (triglyceride) (mg/dL)	Integer	(37, 1050)
42	LDL (low density lipoprotein) (mg/dL)	Integer	(18, 232)
43	HDL (high density lipoprotein) (mg/dL)	Float	(15.9, 111)
44	BUN (blood urea nitrogen) (mg/dL)	Integer	(6–52)
45	ESR (erythrocyte sedimentation rate) (mm/h)	Integer	(1, 90)
46	HB (hemoglobin) (g/dL)	Float	(8.9–17.6)
47	K	Float	(3, 6.6)
48	Na	Integer	(128, 156)
49	WBC (white blood cell) (cells/mL)	Integer	(3700–18,000)
50	Lymph (lymphocyte) (%)	Integer	(7–60)
51	Neut (neutrophil) (%)	Integer	(32–89)
52	PLT (platelet) (1000/mL)	Integer	(25–742)
53	EF (ejection fraction) (%)	Integer	(15–60)
54	RWMA (regional wall motion abnormality)	Integer	(0–4)
55	VHD (valvular heart disease)	Integer	N = 0; mild = 1; moderate = 2; severe = 3
56	Class	Integer	CHD = 1; normal = 0

LBBB, left blood–brain barrier; RBBB, right blood-brain barrier.

Other four datasets include 14 attributes: age, gender, chest pain type, resting blood pressure, serum cholesterol, fasting blood glucose, resting ECG results, acquisition of maximal heart rate, exercise angina pectoris, ST depression, slope of peak exercise ST segments, number of major blood vessels, thalassemias (thal) and cardiac diagnosis.

Patients were classified as having CHD if they had a diameter narrowing greater than or equal to 50%. Otherwise, they were considered normal. As a result, each of the five datasets was divided into two groups: the CHD group and the normal group. The numbers of two groups for each of the five datasets are shown in Table 2 (Ref. [35, 36]).

**Table 2.** **Numbers in the two groups for each of the five datasets**.

Dataset	Sample number of CHD group	Sample number of normal group	Total
Z-Alizadeh Sani dataset [35]	216	87	303
Hungary dataset [36]	106	187	293
Cleveland dataset [36]	139	164	303
Long Beach–Virginia dataset [36]	149	51	200
Switzerland datasets [36]	115	8	123

CHD, coronary heart disease.

### 2.2 Method

Data preprocessing method: For dichotomous data, such as gender (male or female), obesity (yes or no), left ventricular hypertrophy (yes or no), etc., the values are quantized as 1 and 0, respectively. The dataset we collected and used in this study is small and unsuitable for convolutional neural networks. In addition, compared with convolutional neural network (CNN), MLP has fewer layers and fewer training parameters, which requires less time. Some studies have also shown that when the amount of data is small, the performance of the CNN-based and MLP-based models is comparable. Thus, considering the amount of data in this paper and the CNN and MLP characteristics, MLP was used to establish the CHD detection model. For the missing numeric data values, the average of the existing value for the attribute in the category (CHD or normal) is used to complete the missing values. For the missing categorical data values, the integer data within the quantization range of the category were randomly generated. Four datasets, the Hungary dataset, Cleveland dataset, Long Beach–Virginia dataset, and Switzerland datasets, were merged into a mixed dataset. To eliminate inconsistencies, the numeric data in each dataset were normalized to (0,1) and then merged.

Singular value decomposition-based feature selection method: After data preprocessing for missing data filling with average values, features were selected with the SVD method. SVD is an algorithm widely used in machine learning, which has important applications in dimensionality reduction and feature extraction. The basic principle is to decompose a complex matrix into three matrixes: the left singular vector matrix, the singular value matrix, and the right singular vector matrix. The product of these three matrices is equal to that of the original matrix under certain conditions, which provides great convenience for analyzing and processing data.

Any matrix A can be decomposed into the product of three matrices, as shown in Eqn. 1.

(1) $$A=U\,\sum V^{T}$$

where *U* is an orthogonal matrix and is a diagonal matrix.

The singular value matrix is a diagonal matrix in which the diagonal elements are singular values. These values can reflect the important features of the original matrix. By retaining the largest singular values and their corresponding left and right singular vectors, we can reconstruct the original matrix approximately to achieve dimensionality reduction and feature extraction of data. Through SVD decomposition, the dimensionality reduction of the data can be achieved by the largest singular values and their corresponding left and right singular vectors.

SVD can reveal the potential association of CHD features, which helps to improve the accuracy and efficiency of CHD prediction. This study represents the CHD data as a vector matrix, and the SVD method is used for dimensionality reduction and feature extraction. Specifically, we decompose the CHD data matrix using the SVD method to obtain its feature vectors and singular values. Then, the number of principal components to be retained is selected based on the magnitude of the singular values to acquire a downscaled representation of the data. As a result, 10 features were chosen from the original attributes and used to establish the CHD detection model.

**Fig. 2.**
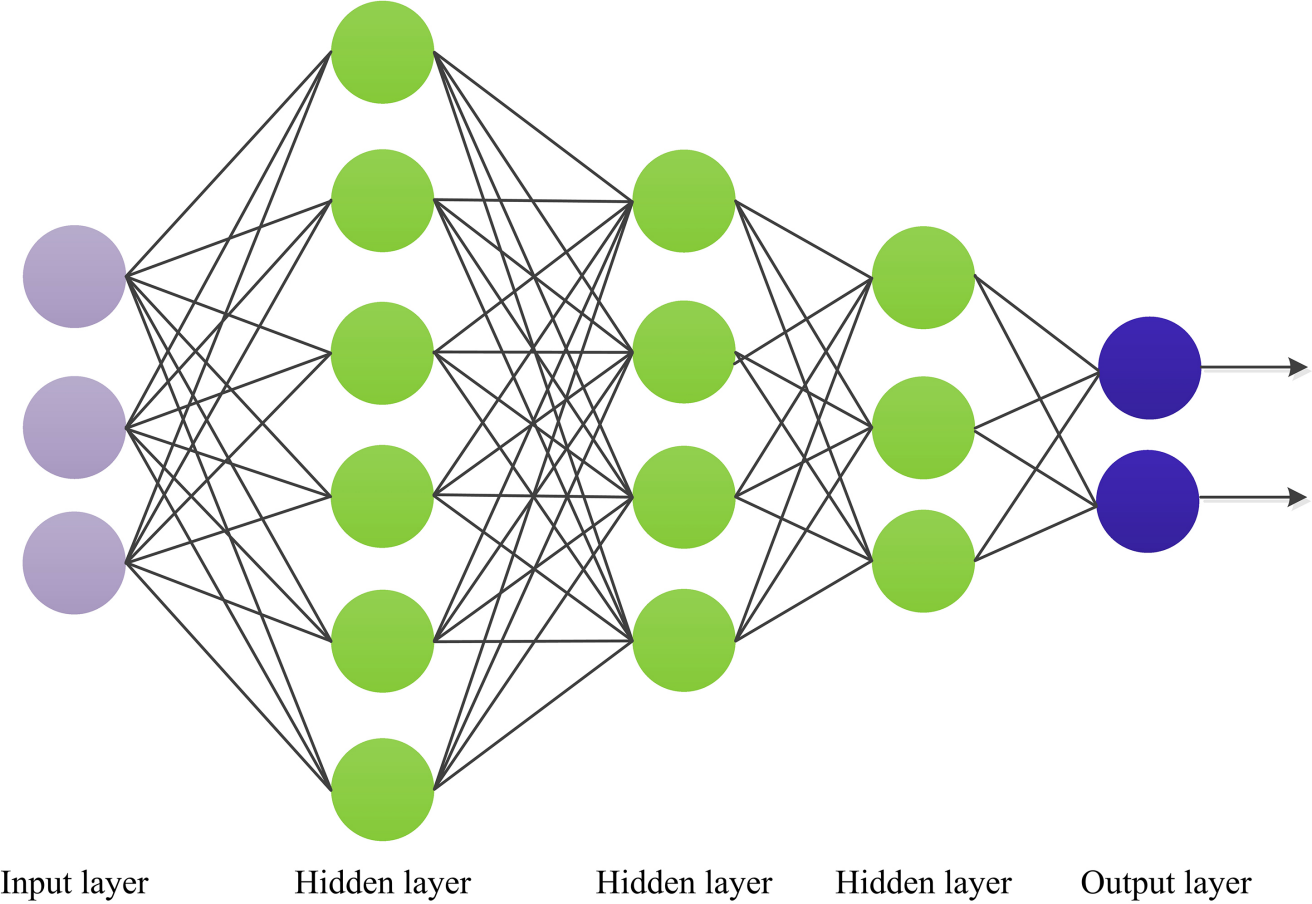
**Schematic diagram for multilayer perceptron (MLP)**.

MLP, an artificial neural network with one input layer, multiple hidden layers, and one output layer, as shown in Fig. 2. The MLP has numerous layers of nodes, each fully connected to the next. In addition to the input nodes, each node is a neuron with a nonlinear activation function. A backpropagation algorithm is used to train MLPs. The MLP method can implement nonlinear discriminant and learn any nonlinear input function. A previous studyfound a nonlinear relationship between clinical factors, such as age, weight, sex, etc., and CHD [22]. The MLP is a universal estimator, which can used to estimate nonlinear functions. Therefore, we used the MLP method in this study to establish the CHD prediction model. The number of features selected determines the number of neurons in the input layer; it was set to 10 in this study. The number of hidden layers was set as 5.

**Fig. 3.**
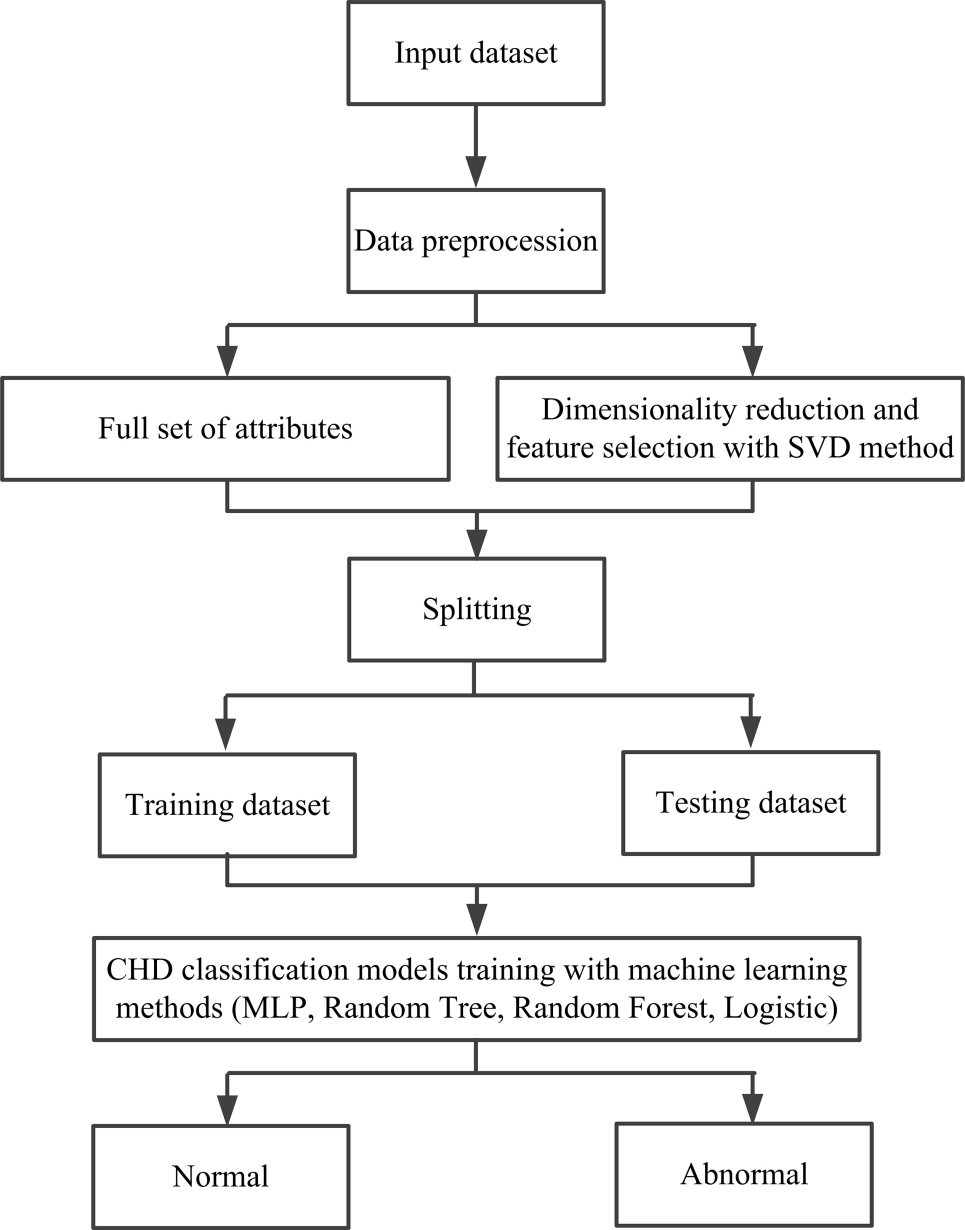
**A flowchart of the established CHD prediction model in this study**. SVD, singular value decomposition; CHD, coronary heart disease; MLP, multilayer perceptron.

CHD prediction model: Since the samples in this study are small, a 5-fold cross-validation method was used to establish the CHD prediction model. Specifically, the stochastic gradient descent method samples were used in the training process, meaning the cross-entropy loss function was applied to optimize the model. The flowchart of this CHD prediction model is shown in Fig. 3. On one hand, in model building, the larger the test set, the smaller the randomness in the measure of model quality, and the more reliable the model performance analysis results are. Due to the small dataset used in this study, a smaller training dataset means a poorer model if we get a large test set by partitioning out more training data. On the other hand, the core of cross-validation is to divide the dataset multiple times and average the results of numerous evaluations, which can eliminate the adverse effects such as overfitting caused by the imbalance of the data in a single partition, and obtain a more reasonable and accurate evaluation of the model. Compared with the traditional model evaluation method (dividing a fixed training set and test set), the advantage of cross-validation is to avoid the problems caused by unreasonable dataset division. The CHD dataset used in this paper is small, meaning it is easy to overfit due to the unreasonable division of the dataset when training the model, so it is more advantageous to use the cross-validation method to evaluate the model. Thus, a 5-fold cross-validation method was used to solve this problem and establish the CHD detection model. Specifically, the normal and diseased samples were divided into five subsets, and then four subsets were used as the training set and the remaining subset as the test set. Then, these were cycled five times until all samples were tested.

### 2.3 Model Evaluation Metrics

Accuracy, precision, recall, *F*_1_-score, and AUC values are used to assess the performance of the CHD prediction model established in this study.

Accuracy is the proportion of samples correctly predicted by the model. The calculation method is shown in Eqn. 2.

(2) $$A\,c\,c\,u\,r\,a\,c\,y=\frac{T\,P+T\,N}{T\,P+T\,N+F\,P+F\,N}$$

Precision is a measure of the ability of the classifier to identify samples correctly; precision expresses the proportion of samples that are correctly predicted out of the samples identified as positive samples, often called the check rate [37]. The calculation method is shown in Eqn. 3.

(3) $$P\,r\,e\,c\,i\,s\,i\,o\,n=\frac{T\,P}{T\,P+F\,P}$$

The recall represents the percentage of all predicted positive samples that can be correctly predicted, usually called the check-perfect rate [38]. The calculation method is shown in Eqn. 4.

(4) $$R\,e\,c\,a\,l\,l=\frac{T\,P}{T\,P+F\,N}$$

In Eqns. 2,3,4, TP is the true positive number. Actual represents a positive sample labeled by professional doctors, and the prediction is a positive sample assessed by model. TP indicates that the classifier correctly predicts positive samples. TN is the true negative number. The actual sample is negative, and the prediction is negative. TN indicates that the classifier correctly predicted a negative sample as a negative sample. FP is the false positive number: an actual negative sample predicted a positive sample. FP indicates that the classifier incorrectly predicted a negative sample as a positive sample, also known as a “false positive”. FN is the false negative number: the actual positive sample predicted the negative sample. FN indicates that the classifier incorrectly predicted a positive sample as a negative sample, also known as a “false negative”.

The *F*_1_-score is a composite evaluation metric, the reconciled average precision rate and recall rate [39], as shown in Eqn. 5.

(5) $$F_{1}=\frac{2\,\cdot \,P\,r\,e\,c\,i\,s\,i\,o\,n\,\cdot \,R\,e\,c\,a\,l\,l}{P\,r\,e\,c\,i\,s\,i\,o\,n+R\,e\,c\,a\,l\,l}$$

The precision and recall rates are calculated using Eqns. 3,4, and then these rates are summed to compute the *F*_1_-score; the higher the *F*_1_-score, the better the performance of the classification model.

## 3. Results and Analysis

Our approach was implemented in a computer environment using the Python programming language and machine learning libraries. The learning rate was set as 0.1, batch size was set as 100, momentum was set as 0.05, and the number of iterations was set as 500.

### 3.1 Performance of CHD Prediction Model Established using Different Machine Learning Methods

To analyze and compare the performance of the CHD prediction model proposed in this study, we also established four other CHD prediction models: MLP without SVD, random tree, random forest, and logistic; the results are shown in Table 3.

**Table 3.** **Performance comparison between the methods proposed in this study and the classic machine learning method using the Z-Alizadeh Sani dataset**.

Method	Accuracy	Precision	Recall	*F*_1_-score	AUC
Random tree	86.47%	86.40%	86.50%	86.40%	83.30%
Random forest	91.75%	91.70%	91.70%	91.70%	97.00%
Logistic	94.39%	94.40%	94.40%	94.40%	98.20%
MLP without SVD	86.14%	86.90%	86.10%	86.40%	90.90%
MLP with PCA	93.40%	93.50%	93.40%	93.40%	97.70%
Proposed method (MLP with SVD)	94.39%	94.50%	94.40%	94.40%	98.20%

MLP, multilayer perceptron; SVD, singular value decomposition; PCA, principal component analysis; AUC, area under the curve.

From Table 3, we can see that compared to the other three models established using SVD-based selection features, our model achieved the best performance with an accuracy of 94.39%, a precision of 94.5%, a recall of 94.4%, *F*_1_-score of 94.4%, and AUC of 98.2%. The AUC values for our proposed model were 13.7% and 1.2% higher than the random tree-based and random forest-based models, respectively, and the same versus the logistic-based model. In addition, we also compared the performance of models established using the MLP method with all features, features selected with the PCA method, and features selected with the SVD method. The results showed that the performance of the SVD-based features method was better than that of the other two. The accuracy of the SVD-based model was 7.3% and 0.5% higher than the all-features- and PCA-based models, respectively.

**Fig. 4.**
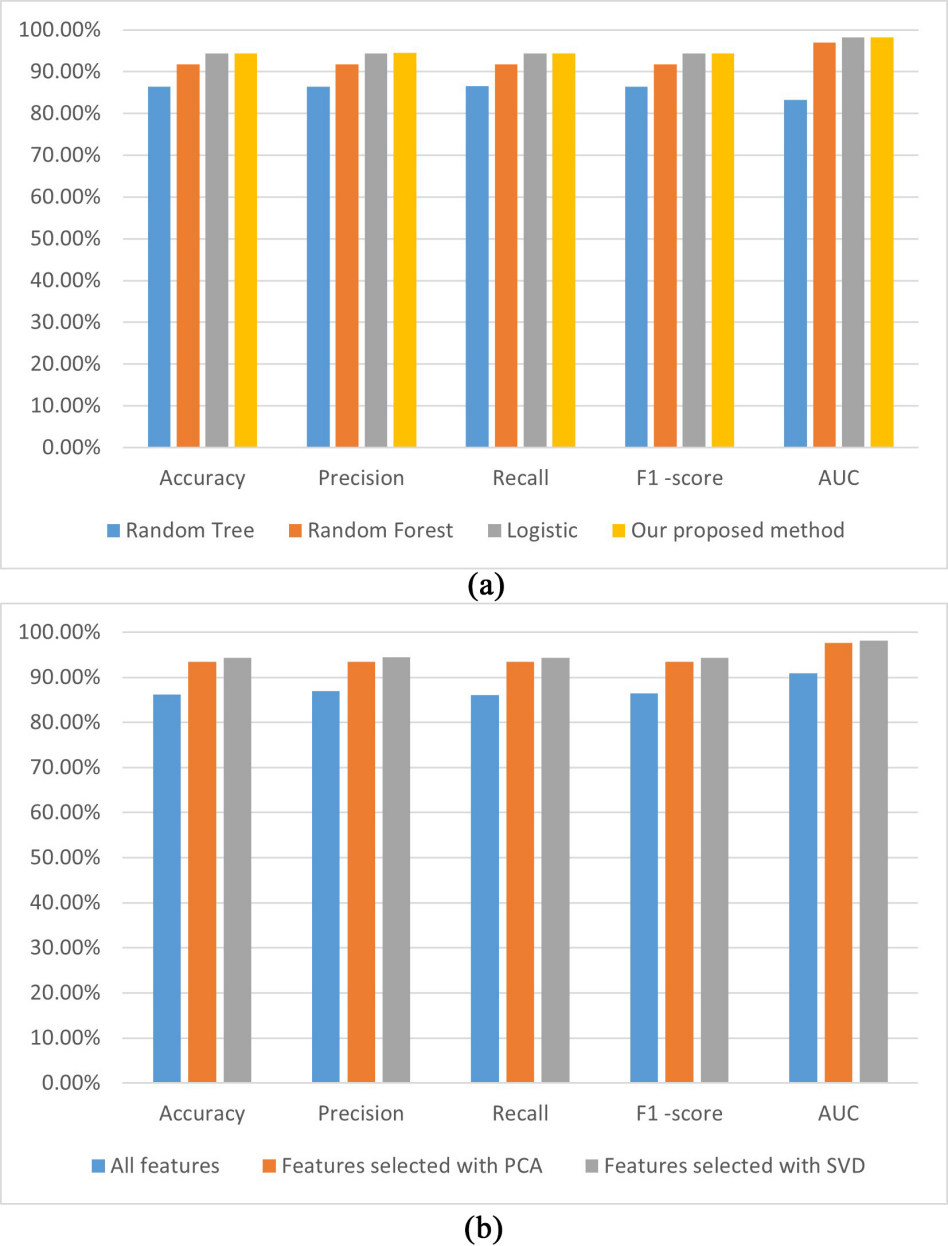
**The performances of the established CHD prediction models with different classifiers using the SVD-selected features (a); the models built with the MLP using different features (b)**. AUC, area under the curve; PCA, principal component analysis; SVD, singular value decomposition; CHD, coronary heart disease; MLP, multilayer perceptron.

The visualization performances of the different models established in this study are shown in Fig. 4, whereby Fig. 4a shows the performance of models established with SVD-based features using random tree, random forest, and logistic, respectively. Fig. 4b shows the performances of the models built using the MLP method with all PCA- and SVD-based features, respectively.

Fig. 4a illustrates that the accuracy, precision, recall, *F*_1_-score, and AUC performances of our proposed model are the highest among the analyzed models. A possible reason is that the MLP method can learn the nonlinear function of the input and implement nonlinear discriminant. Fig. 4b demonstrates that all performance indicators for our proposed model are higher in the three models established with different features. A possible reason is that SVD can reveal the potential association of attributes and can improve the performance of the CHD prediction model.

**Fig. 5.**
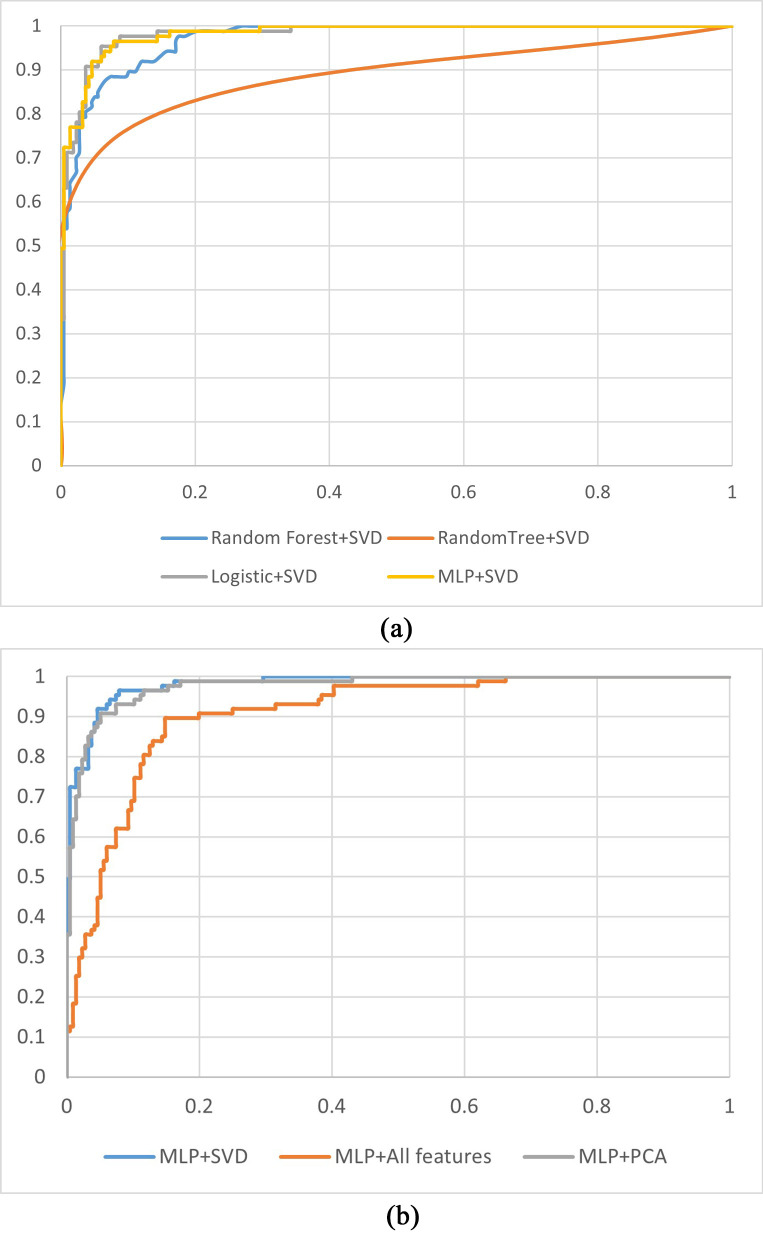
**ROC curves for the CHD prediction models**. (a) ROC curves for models established with different machine learning methods using features selected for the SVD method. (b) ROC curves for models established with the MLP using different features. PCA, principal component analysis; SVD, singular value decomposition; CHD, coronary heart disease; MLP, multilayer perceptron; ROC, receiver operating characteristic.

Fig. 5 shows the ROC curves for the CHD prediction models. Fig. 5a shows the receiver operating characteristic (ROC) curves of the models established with different classifiers using SVD-selected features. Fig. 5a illustrates that the ROC curves of the MLP + SVD-based and logistic + SVD-based models are close to coincident, which means no significant difference occurred in the AUC values between the two methods. The AUC value of the MLP + SVD-based model is higher than those for the random forest + SVD and random tree-based models. Fig. 5b shows the ROC curves of the models built with a classifier of the MLP using features selected for the SVD method and all features and features selected for the PCA method. From Fig. 5b, we can see that the AUC value of the SVD-selected features is higher than for all features-based and PCA-based models.

Table 4 shows the confusion matrix computed using the prediction score of the proposed model. The overall prediction accuracy of the proposed model was 94.4%; that is, 286 of the 303 patients were correctly classified, while 5.61% (17/303) patients were misclassified, which corresponds to an 88.89% (80/90) prediction ‘sensitivity’ with a 96.71% (206/213) prediction ‘specificity’. The positive predictive value (PPV) of the risk model was 91.95% (80/87), and the negative predictive value (NPV) was 95.37% (206/216).

**Table 4.** **Confusion matrix for the Z-Alizadeh Sani dataset**.

	Positive	Negative
Positive	80	7
Negative	10	206

We performed the Hosmer–Lemeshow test and calculated a *p*-value of 0.162 and a chi-square of 7.902, which indicates the effectiveness of the proposed model performs well.

For each sample, there is one disease prediction. A total of 303 predictions were computed using the leave one case out (LOCO) method. These predictions were divided into three groups, with 101 sample predictions in each group, and the number of diseased and non-diseased samples in each group was calculated. The first group was used as the baseline, and the odds ratio value and the corresponding 95% confidence interval values were calculated, as shown in Table 5.

**Table 5.** **Adjusted odds ratios (ORs) and 95% confidence intervals (CIs) for three bins with increasing risk scores**.

Bins	CHD–normal sample numbers	Adjusted odds ratios	95% confidence intervals
1	68–33	1	Baseline
2	71–30	1.1485	(0.6329, 2.0841)
3	77–24	1.5570	(0.8386, 2.8907)

CHD, coronary heart disease.

As shown in Table 5, the odds ratio (OR) values increased from 1 to 1.1485 and then to 1.5570, which means the CHD prediction scores increased as the cases were determined to have CHD.

**Fig. 6.**
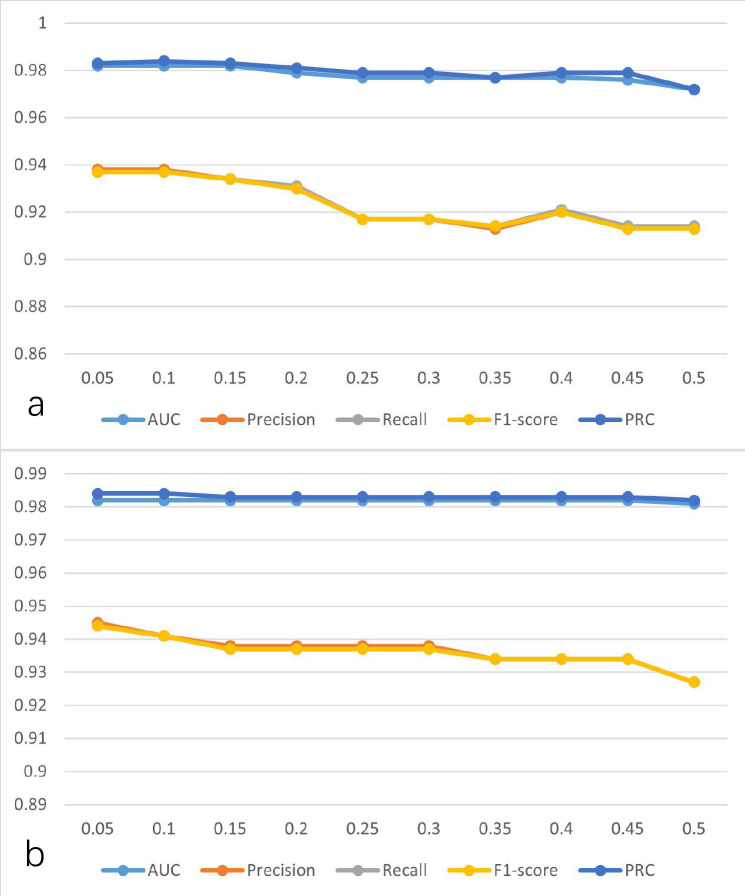
**The performance of the model was established using SVD-selected features with the MLP at different parameters**. (a) Learning rate at different values. (b) Momentum at different values. AUC, area under the curve; PRC, precision-recall curve; SVD, singular value decomposition; MLP, multilayer perceptron.

To explore the influence of hyperparameters on the performance of the model, the learning rate was gradually increased from 0.05 to 0.5 at intervals of 0.05, and the changes in model performance indicators are shown in Fig. 6a. As illustrated in Fig. 6a when the learning rate is set to 0.1, the performance of the model is better than others. Similarly, the performance indicators are shown in Fig. 6b for when the momentum is gradually increased from 0.05 to 0.5 at intervals of 0.05. As can be seen from Fig. 6b, when the momentum is set to 0.05, the performance of the model is better than others. Thus, the learning rate and the momentum were set to 0.1 and 0.05, respectively.

### 3.2 Performance Validation of CHD Prediction Model on a Mixed Dataset

**Fig. 7.**
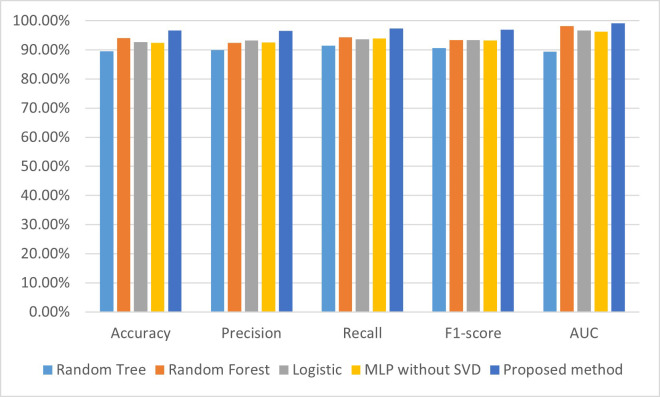
**Comparison of performances for CHD detection models on a mixed dataset**. AUC, area under the curve; SVD, singular value decomposition; CHD, coronary heart disease; MLP, multilayer perceptron.

To compare and analyze the performance and robustness of our proposed method, we tested our method on a constructed dataset that mixed with four well-known public cardiology datasets: Hungary, Cleveland, Long Beach–Virginia, and Switzerland, which contained 293, 303, 200, and 123 samples, respectively. Moreover, the samples in each of the four datasets have 14 attributes. Thus, 920 cases are contained in this mixed dataset, each with 14 attributes. After data preprocessing, which comprised filling missing data and normalization, the SVD method was applied to extract features from this mixed dataset. Then, machine learning methods, including random tree, random forest, logistic, and our MLP, were used to establish the CHD detection model. In addition, we used all attributes without SVD to construct the CHD detection model; the results are shown in Table 6, and visualization of these results is shown in Fig. 7.

**Table 6.** **A comparison of the performances with CHD detection models was established using different machine learning methods on a hybrid dataset consisting of four independent datasets**.

	Accuracy	Precision	Recall	*F*_1_-score	AUC
Random tree	89.57%	89.90%	91.40%	90.60%	89.40%
Random forest	94.02%	92.40%	94.40%	93.40%	98.20%
Logistic	92.72%	93.20%	93.70%	93.40%	96.70%
MLP without SVD	92.39%	92.50%	93.90%	93.20%	96.30%
Proposed method	96.63%	96.50%	97.40%	97.00%	99.10%

AUC, area under the curve; SVD, singular value decomposition; CHD, coronary heart disease; MLP, multilayer perceptron.

From Table 6, we can see that the accuracy, precision, recall, *F*_1_-score, and AUC of our proposed model was 96.63%, 96.5%, 97.4%, 97%, and 99.1%, respectively, which are all higher than the models established using random tree, random forest, logistic, and the MLP without SVD. Specifically, the accuracy, precision, recall, *F*_1_-score, and AUC of our proposed model are higher by 2.61%–7.06%, 3.3%–6.6%, 3%–6%, 3.6%–6.4%%, and 0.9%–9.7%, respectively, than the other four CHD prediction models established in this study.

From Fig. 7, we can see that the performance indicators for the accuracy, precision, recall, *F*_1_-score, and AUC of our proposed model are the highest of the five CHD prediction models. The experimental results show that the SVD and MLP-based model performs well in predicting heart disease, outperforming the other methods in accuracy, recall, and *F*_1_ values, and has a higher predictive performance. This indicates that SVD can extract important features from the data and input them into the MLP model for effective classification and prediction.

Table 7 shows the confusion matrix calculated using the prediction score of the proposed model on the mixed dataset. The accuracy of the proposed model was 96.63%; that is, 889 of the 920 cases were correctly classified, while 3.37% (31/920) cases were misclassified. Moreover, the sensitivity and specificity values were 96.8% (393/406) and 96.5% (496/514), respectively. The PPV of the risk model was 95.62% (393/411), and the NPV was 97.45% (496/509).

**Table 7.** **Confusion matrix on a hybrid dataset**.

	Positive	Negative
Positive	393	18
Negative	13	496

### 3.3 Analysis of SVD Attribution for CHD Prediction

**Fig. 8.**
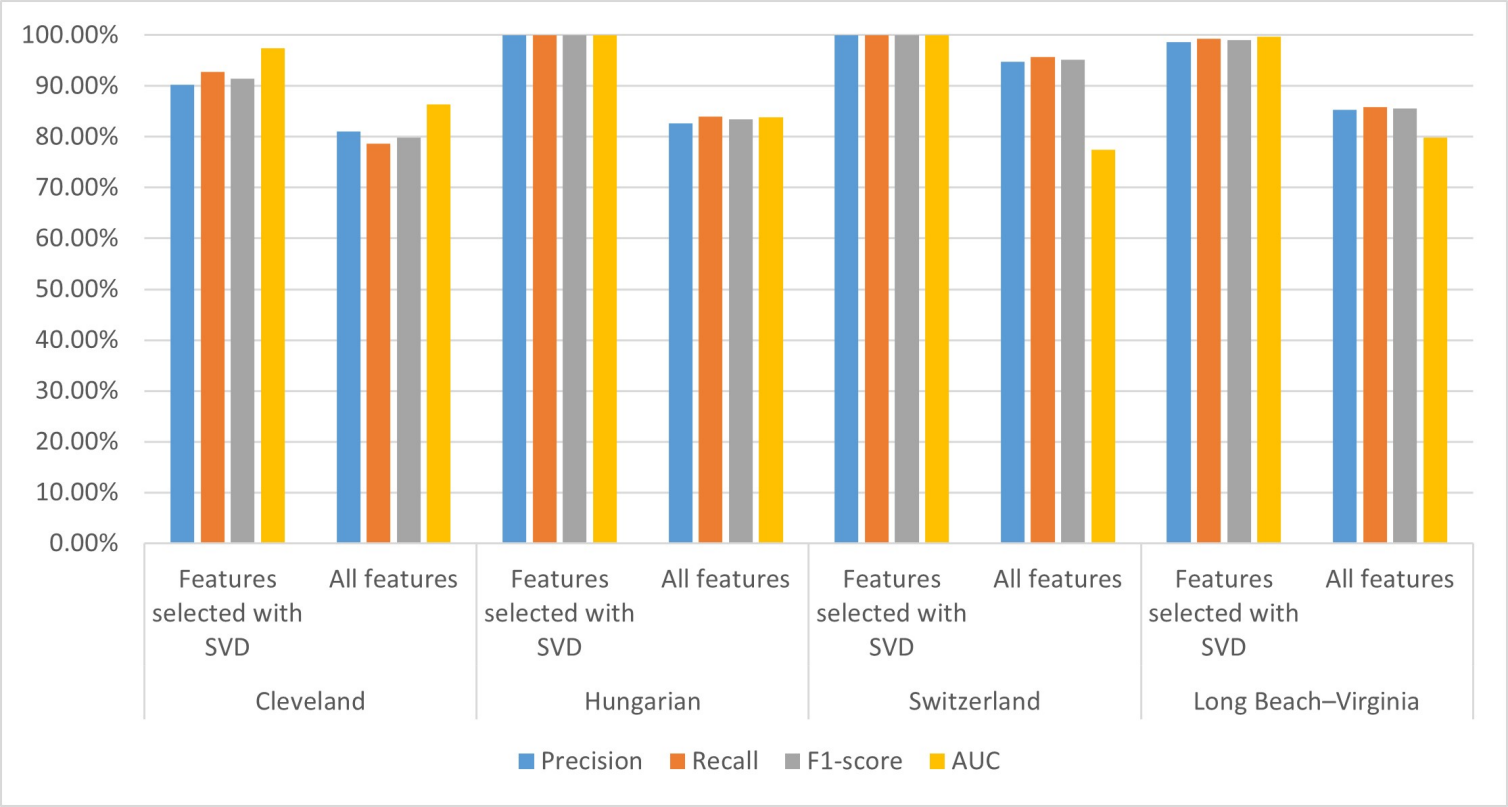
**Performance of the CHD prediction model on different datasets**. Performances for the Cleveland, Hungary, Switzerland, and Long Beach–Virginia (Long beach–va) datasets. AUC, area under the curve; SVD, singular value decomposition; CHD, coronary heart disease.

To analyze and compare the SVD method contribution to the performance of the CHD prediction model, we established the CHD prediction model with all 14 attributes and features extracted by the SVD method and tested it on four well-known cardiology datasets. The performances of the CHD prediction models on the different datasets are shown in Table 8; visualization of these results is shown in Fig. 8.

**Table 8.** **A performance comparison of four datasets between established models with different features**.

Datasets	Methods	Precision	Recall	*F*_1_-score	AUC
Cleveland	Features selected with SVD	90.20%	92.80%	91.50%	97.50%
	All features	81.10%	78.70%	79.90%	86.40%
Hungarian	Features selected with SVD	100.00%	100.00%	100.00%	100.00%
	All features	82.70%	84.00%	83.40%	83.80%
Switzerland	Features selected with SVD	100.00%	100.00%	100.00%	100.00%
	All features	94.80%	95.70%	95.20%	77.50%
Long Beach–Virginia	Features selected with SVD	98.70%	99.30%	99.00%	99.70%
	All features	85.30%	85.90%	85.60%	79.90%

AUC, area under the curve; SVD, singular value decomposition.

From Table 8, we can see that the performance of the CHD detection model established using SVD-based selected features is better on each of the four datasets than the model built using all features. Specifically, the AUC values of our proposed model achieved 97.50%, 100.00%, 100.00%, and 99.70% on the Cleveland, Hungarian, Switzerland, and Long Beach–Virginia datasets, respectively, which is 11.1%, 16.2%, 22.5%, and 19.8% higher, respectively, than the model built using all features. The precision, recall, and *F*_1_-score values of our proposed model on the four datasets are 5.2%–17.3%, 4.3%–16%, and 4.8%–16.6% higher, respectively, than the model built using all features.

From Fig. 8, we can see that for each of the four datasets, the performance indicators of our proposed model are all higher than the model built with all features. These results indicate that introducing the SVD method for feature extraction and further model construction helps improve its performance. This may be due to their existing redundancy and correlation between attributes in the dataset that affect the performance of the model. The SVD method can reduce dimensions and extract data features. In addition, the potential association between characteristics of the CHD dataset can be revealed using the SVD method, thus helping to improve the performance of the model.

## 4. Discussion

Coronary heart disease is one of the leading causes of death worldwide, affecting health and causing a significant global burden. Therefore, it is essential to diagnose CHD at an early stage to reduce health risks and prevent cardiac arrest. Various data such as medical imaging, gene expression data, biomarkers, and clinical information can be used to predict CHD. There are also limitations for different types of data. For example, the genetic information-based CHD prediction method requires large-scale genetic data, and the biomarkers-based method remains challenging to interpret and apply generally. Furthermore, the medical imaging-based method demands expertise and highly complex algorithms. Hence, developing an automated CAD framework for accurate CHD diagnosis is important. Clinical data-based methods have been widely discussed due to their effectiveness and speedability. Meanwhile, accuracy and robustness are required to improve and satisfy clinical application requirements. This study developed a novel, improved CHD prediction model by training the multilayer perceptron using features selected for the SVD method, which is applicable in real-time since it applies to different clinical attributes and provides fast computation. This study has several unique characteristics, as follows.

First, we proposed a real-time, applicable, clinical information-based CHD detection model by combining the SVD and MLP methods. The results show that our proposed method has an accuracy of 94.39%, a precision of 94.5%, a recall of 94.4%, *F*_1_-score of 94.4%, and an AUC of 98.2% when using the Z-Alizadeh Sani dataset, which were all higher than for the other four CHD prediction models established in this study.

Results were analyzed using the CHD detection models from previous studies to compare further and explore our CHD prediction model. As shown in Table 9 (Ref. [20, 21, 23, 24, 25, 26, 28, 29, 30, 31]), our proposed model achieved accuracy and AUC values of 94.39% and 98.2%, respectively. In comparison, the AUC of the four models mentioned above was 10%, 11.2%, 11.45%, and 2.1% lower, respectively, than our proposed model in this study. Additionally, the accuracy of eight of the ten abovementioned models was lower than our proposed model. The accuracy of the two models achieved 99.1% and 98.15% in previous studies. A possible reason is that the deep neural network was used, which has more parameters and computational quantities [20, 30].

**Table 9.** **Comparative results using the CHD detection models from previous studies**.

References	AUC	Accuracy
Amarbayasgalan *et al*. [21]	88.2%	89.2%
Ananey-Obiri and Sarku [23]	87%	82.75%
Napa *et al*. [24]	86.75%	85.71%
Trigka and Dritsas [28]	96.1%	
Mohan *et al*. [26]		88.7%
Enad and Mohammed [29]		85%
Perumal and Kaladevi [25]		87%
Sarra *et al*. [31]		89.7%
Ayon *et al*. [20]		98.15%
Sarra *et al*. [30]		99.1%
Our Proposed model	97.70%	93.07%

AUC, area under the curve; CHD, coronary heart disease.

Second, the proposed model allows feature vectors of different sizes to overcome the challenge of data size changes with clinical information. In addition, the performance of the model trained using features selected with SVD is better than that of those trained using all clinical information. We also compared the performance of different machine learning-based model training using a hybrid dataset fused with four public CHD datasets. These results indicated that our proposed method has an accuracy of 96.63%, a precision of 96.5%, a recall of 97.4%, *F*_1_-score of 97%, and an AUC of 99.1% on this mixed dataset; all are higher than for the other four CHD prediction models.

Third, the proposed intelligent system uses the SVD method and multilayer perceptron, thereby eliminating existing deep-learning model limitations. The SVD is a matrix decomposition method that can decompose a matrix into its principal components and corresponding weights. This method is especially useful when working with high-dimensional data, as it helps identify the main information and structure in the data. In terms of feature selection, SVD can be used to select those features that contribute the most to the performance of the model to reduce the number of features and improve the accuracy and efficiency of the model. “Simple matrices” are used to represent “complex matrices” linearly, and these “simple matrices” happen to be the results of the tensor product of their corresponding eigenvectors. Reasons for using the SVD method for feature selection are: (1) compared with the CNN-based feature selection method, the SVD-based feature selection method has fewer parameters, lower computation, and higher efficiency than the CNN method. (2) The dataset in this paper is small and does not apply to CNN. (3) The PCA-based feature selection method establishes the CHD detection model. These results show that the CHD detection model established using SVD-based selection features achieved more than all features- and PCA-based selection features in the Z-Alizadeh Sani dataset, which indicates that the SVD method can improve the performance of the prediction model compared to traditional feature selection methods. On the one hand, the MLP is suitable for building machine learning models with a few features, whereas convolutional neural networks require a large amount of data to be trained to demonstrate their advantages. On the other hand, the datasets we collected and used in this study are small and, thus, unsuitable for convolutional neural networks. In addition, compared with CNN, the MLP has fewer layers and fewer training parameters, which requires less time. Some studies have also shown that when the amount of data is small, the performance of the CNN-based and MLP-based models is comparable [4, 5]. Thus, considering the amount of data in this paper and the CNN and MLP characteristics, the MLP was used to establish the CHD detection model.

As a result, the developed CHD detection model represents a highly effective work that applies the clinical information with a machine learning-based method, making it faster and better on multiple datasets than other CHD detection models. In addition, the developed CHD detection model will aid medical professionals in performing diagnoses quickly and prioritizing their work. Overall, the SVD and MLP-based heart disease prediction model outperforms the other methods. Further, the SVD and MLP-based heart disease prediction model can extract important features from high-dimensional data and perform nonlinear modeling using the multilayer perceptron, thereby improving the accuracy and reliability of the prediction.

However, there are three important potential limitations in this study. First, although five datasets were used, the sample size was small, which may affect the generalizability of the model. Thus, we need to collect and use more samples to validate the generalization ability and stability of the model on other datasets. Second, SVD may corrupt the sparsity of the original data, resulting in the loss of some important information. Furthermore, SVD is less interpretable. Thus, a more efficient feature extraction method must be developed to establish a high-performance CHD predictive model. Third, although the MLP can solve complex nonlinear classification problems, the MLP has limitations in sensitivity to parameter selection and falls into the local optimal solution rather than the global solution. Hence, better classifiers need to be studied further.

## 5. Conclusions

The incidence of CHD in both men and women increases with age, which seriously affects the quality of life of patients; hence, building a highly accurate and efficient predictive model for CHD is important. In this study, we developed a novel heart disease prediction model based on the SVD and MLP methods, demonstrating improved performance on different datasets.

The accuracy and AUC for a novel model were 0.94.4% and 98.2%, respectively, with 0.24 seconds on the Z-Alizadeh Sani dataset, which has 303 patients and 55 attributes. Moreover, the accuracy and AUC reached 96.63% and 99.1%, respectively, when using a hybrid dataset comprising four independent datasets. In addition, this proposed model allows different sizes of attributes, which overcomes the challenge of irregularity in the data shape of clinical information. The application potential of the model will contribute to early intervention and personalized treatment, ultimately improving the quality of life and health status of patients. Through this study, we hope to provide new perspectives and methods for research and application in heart disease prediction.

Some potential limitations include a small dataset, poorly interpretable SVD method, and high parameter sensitivity of the MLP. In the future, we will collect more CHD-related data and develop highly interpretable feature extraction methods and high-performance and robust classifiers to build interpretable CHD prediction models with improved performances.

## Availability of Data and Materials

The data presented in this study are available on request from the corresponding author.
